# Microfabricated Reference Electrodes and their Biosensing Applications

**DOI:** 10.3390/s100301679

**Published:** 2010-03-02

**Authors:** M. Waleed Shinwari, David Zhitomirsky, Imran A. Deen, P. R. Selvaganapathy, M. Jamal Deen, D. Landheer

**Affiliations:** 1 McMaster University, Hamilton, ON L8S 4K1 Canada; E-Mails: shinwamw@univmail.cis.mcmaster.ca (M.W.S.), zhitomd@muss.cis.mcmaster.ca (D.Z.), deenia@univmail.cis.mcmaster.ca (I.A.D.), selvaga@mcmaster.ca (P.R.S.); 2 IMS, National Research Council, Ottawa, ON, Canada; E-Mail: dolf.landheer@nrc.ca

**Keywords:** electrode, reference electrode, microfabrication, biosensor, electrochemical, lab-on-chip

## Abstract

Over the past two decades, there has been an increasing trend towards miniaturization of both biological and chemical sensors and their integration with miniaturized sample pre-processing and analysis systems. These miniaturized lab-on-chip devices have several functional advantages including low cost, their ability to analyze smaller samples, faster analysis time, suitability for automation, and increased reliability and repeatability. Electrical based sensing methods that transduce biological or chemical signals into the electrical domain are a dominant part of the lab-on-chip devices. A vital part of any electrochemical sensing system is the reference electrode, which is a probe that is capable of measuring the potential on the solution side of an electrochemical interface. Research on miniaturization of this crucial component and analysis of the parameters that affect its performance, stability and lifetime, is sparse. In this paper, we present the basic electrochemistry and thermodynamics of these reference electrodes and illustrate the uses of reference electrodes in electrochemical and biological measurements. Different electrochemical systems that are used as reference electrodes will be presented, and an overview of some contemporary advances in electrode miniaturization and their performance will be provided.

## 1. Introduction

Recently, there has been growing interest in the development of miniaturized sensors for biomedical and environmental applications, both for monitoring biologically significant analytes and environmental conditions such as pH, physiological markers such as oxygen and carbon dioxide content in blood, as well as detection of pathogenic bacteria and viruses. This interest has been in response to the increasing need to prevent outbreaks and cross contamination in the global food supply and processing chain and maintenance of safe drinking water supply, as well as in medical settings where the patient’s physiological condition has to be monitored in real-time.

Although a wide array of biosensors based on optical, electrical, piezoelectric, resonant and thermal transduction for biomedical and environmental applications exists, electrochemical biosensors are some of the simplest, low-cost, reliable and practical sensors [1–4]. Electrochemical biosensors for biomedical and environmental applications have been developed employing various fabrication methods such as photolithography, screen-printing and ink-jet printing. In all these biosensors, the electrochemical transducer, which converts the quantity and type of chemical/biological analyte into a distinct electrical signal, is the most important component. Typically, the transduction results in either a change in voltage at an electrode interface or a flow of charge when the potential of the electrode is maintained constant relative to the solution. A crucial component that measures and controls the solution side potential is the reference electrode. The reference potential set by the reference electrode must be insensitive to any changes occurring in the electrolyte solution and must maintain a constant potential throughout. The current trend towards miniaturization of the biosensors for higher speed, performance and portability has meant miniaturization of not just the sensing element, but also of the reference electrode.

Reference electrode miniaturization has several implications which require considerable attention. All components of the electrode such as metal, salt, filling solutions and interfaces need to be miniaturized. Factors such as potential drift, liquid junction effects and solution concentrations are significant factors which could easily obscure correct results and need to be controlled [5,6]. Thus, in constructing such electrodes, there are several factors which are needed to be taken into account [6]:

1. The reference electrodes must have high exchange current densities and thus be non-polarizeable (hard to change the electrode’s potential).
2. The potentials of all of the electrodes must be reproducible.
3. The potential drift of the electrodes, from factors attributed to filling solution effusion or degradation of electrode coating, must be minimized over the duration of device operation.
4. Other operational requirements needed for the working electrodes might be imposed based on the nature of the experiment. For example, invasive recording and stimulating electrodes should not introduce foreign toxic ions into humans, which necessitate the use of highly polarizeable electrodes.

The reference electrode has a pronounced influence on the accuracy of measurements made with such bio-sensors, whether they are electrochemical sensors, biological Field Effect Transistor (bio-FET) [7,8] or bio-impedances probes [9,10], as it is present in almost all types of biosensors, and plays an equally important role in ensuring proper device operation and reliability of the results. Evidently, in order to facilitate for the development and successful operation of an array of miniaturized sensors, it is vital to fabricate functional and robust miniaturized reference electrodes.

This review is organized as follows. In the following section, we describe the basic dynamic operation of electrodes, and this will lead us to establishing criteria for the design of reference electrodes. Then, some common types of reference electrodes will be presented, followed by sections on miniaturized and microfabricated reference electrodes and their performance. Finally, examples of common uses of electrodes in chemical and biological sensing will be given.

## 2. Basic Theory

### 2.1. Electrode Potential

Any electrochemical interface consists of an electrode (typically metal) and an electrolyte with a potential associated with the electrode. The potential represents the energy per unit charge required to take a charge from one side of the interface to the other. This electrode potential can be related to the chemical activities of the constituents of the electrochemical reaction by the Nernst Equation.

(1) $$E_{\mathrm{red}}=E_{\mathrm{red}}{}^{0}-\frac{\mathrm{RT}}{zF}\text{ln}\left(\frac{a_{\mathrm{red}}}{a_{\mathrm{ox}}}\right)$$

where *E* is the electrode reduction potential, *E^o^_red_* is the standard reduction potential, *R* is the universal gas constant, *T* is the temperature in Kelvin, *z* is the number of electrons transferred per reaction, *F* is Faraday’s constant, and *a_red_* and *a_ox_* denote the chemical activity of the reduced and oxidized species, respectively. Electrode potentials are often expressed in terms of half-cell potentials with respect to the standard hydrogen electrode (SHE) which has a conventional potential of zero.

### 2.2. Current-Voltage Relationship

When an electrode-electrolyte interface is at its standard potential, it is at equilibrium and no net reaction occurs at the interface. With current flow due to application of an external potential, this thermodynamic equilibrium is violated, and one must resort to kinetic models to be able to characterize the electrochemical system under non-equilibrium conditions. There are two major factors influencing this response. In solution, the electrolysis of the analyte depends on the transfer of electrons between the electrode and substituents at the interface, and replenishment of interfacial analyte once it has been electrolyzed [12,13]. At low applied potentials and currents, electron transfer is predominantly dependent on standard redox potential and applied potentials. Most of the applied potential occurs across the interfacial regions, and the electrochemical equations ultimately determine the current flow through the rates of reaction. The rate of the chemical reaction will depend on the applied potential and the electrode potential at zero bias. A simple model for the current-voltage characteristics is given by the Butler-Volmer equation:

(2) $$j=j_{0}\left(e^{\frac{\alpha zq\eta }{kT}}-e^{\frac{-(1-\alpha )zq\eta }{kT}}\right)$$

where:

*j* is the current density,
*η=V*−*V_eq_* is the “overpotential”, defined as the difference between the applied potential and the built-in potential *V_eq_* of the electrode,
*α* is the symmetry factor, which is a linearization coefficient for the effect of the overpotential on the energetics of the reaction,
*k* is Boltzmann’s constant,
*q* is the elementary charge, and
*j_0_* is the exchange current density.

This equation shows that, for a kinetically controlled process, the flow of charges across the electrode electrolyte interface will induce a change in potential of the electrode. The exchange current density is the key determining factor in the magnitude of the change. The higher the exchange current density, the lower is the change in potential. Thus, higher exchange current density in the reaction system is sought for use as reference electrodes and such systems are said to be well poised. The Butler-Volmer equation is only accurate for the so-called outer-sphere reactions. In such reactions, the only process that happens is electron tunneling between the electrode and the analyte, with no intermediate step such as specific adsorption or intermediate reactions. An example of an outer sphere reaction would be the hydrogen evolution on platinum, which is used in the standard hydrogen reference electrode. Unfortunately, not all reference systems are outer sphere reactions. For example, Ag/AgCl reaction does not fall into this category. However, Equation (2) can still provide useful insight into the design parameters for the electrode. The exchange current density is given by:

(3) $$j_{0}=qK{\left(n_{\mathrm{red}}^{s}\right)}^{1-\alpha }{\left(n_{\mathrm{ox}}^{s}\right)}^{\alpha }e^{\frac{-\Delta G^{0}}{kT}}$$

where *n_red_^s^* and *n_ox_^s^* are the concentrations of the reduced and oxidized species per unit area, respectively, Δ*G*^0^ is the activation energy for the forward and reverse reactions at equilibrium, and *K* is the pre-exponential factor which is proportional to the collision rate between reacting species and depends on the geometry and mass of the molecules, and on the temperature. The higher the exchange current density, the smaller is the electrode-electrolyte interfacial resistance and the more stable is the electrode potential. The exchange current densities for many different electrode reactions are summarized in [14,15].

**Figure 1.**
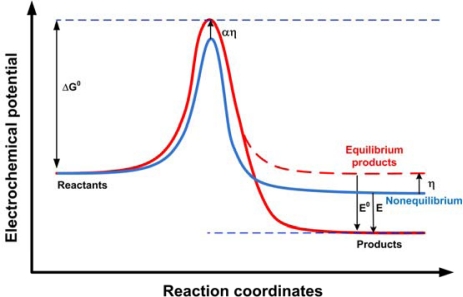
Outer-sphere reaction effect on the reaction potentials.

Figure 1 shows a potential diagram explaining the outer-sphere electron-transfer reactions. Upon electrode-electrolyte contact, spontaneous reactions occur due to the electrochemical potential difference. When this reaction is at equilibrium, the reactants and products have the same electrochemical potential (dashed line), with an accompanying *E_0_* electrostatic energy change. Now, if an external source of energy is introduced to the system, the electrostatic potential of the products can be reduced from *E_0_* to *E*, and the reaction can be made to proceed. The applied potential *η* affects the height of the barrier for the reaction as well, which gives rise to the exponential terms in the Butler-Volmer equation.

At low applied voltages, the reaction barrier (Figure 1) determines the rate of current transfer. However, at higher applied voltages, this reaction barrier becomes of less significance, and the current-determining phenomenon is the rate of transport of the reacting species to the electrodes, known as mass transport. Mass transport can be achieved via migration, convection, or diffusion [12]. Diffusive flow is spontaneous and occurs due to concentration gradients that build up as a result of fast consumption of reactants at the interfaces. Convective flow is the mechanical “dragging” of reactants by the hydrodynamic flow of the bulk electrolyte, due to viscous forces. Migration is the transport of charged reactants by electrostatic attraction to a charged electrode. Diffusion can occur in both directions: towards the electrode and away from it. When the reaction rate is fast enough, depletion of the reacting species at the interface causes diffusion of new reactants from the bulk, which can place an upper limit on the rate of the reaction and current flow. Similarly, abundance of the products can inhibit the progress of the reaction due to their effect on the chemical potential of the reacting species. If the reaction results are dissolved ions, then their diffusion away from the bulk might be the current limiting factor.

### 2.3. Reference Electrodes

The reference electrode, a specific type of electrode comprised of well-defined materials, has a known stable potential to which all other electrode potentials are referenced. For example, the potential of the Standard Hydrogen Electrode (SHE) at standard conditions (25 °C, 1 bar pressure and HCl solution with activity of 1 mol/kg) is fixed as zero. However, due to its complexity it is not used as often. The Ag/AgCl electrode is arguably the simplest and most practical reference electrode used in industry and research. Its simple construction, consisting of Ag metal coated with AgCl immersed in a saturated filling solution, and cheap manufacturing costs, make it an attractive choice.

**Figure 2.**
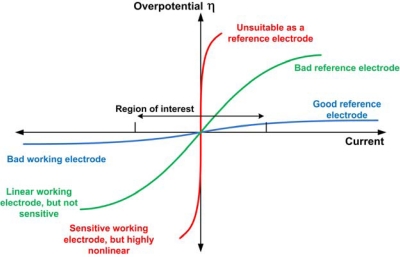
Symmetric I–V curves for electrodes of varying polarizability.

The requirements for a reference electrode can be summarized as follows:

1. The reference electrode must have a high exchange current density, must be reversible and non-polarizeable. These properties will allow exchange of charge between the electrode-electrolyte interface due to electrochemical reactions and other environmental factors without significantly changing the electrode potential. It will also guard against fluctuations caused by random charge injection into the interface. Figure 2 shows typical current-voltage profiles, indicating the range of good reference electrodes.
2. The electrode reaction should not consume the electrode or change its surface area in any way, so as not to change the reaction area and hence the current.
3. The solution in contact with the reference electrode should be saturated. There are many reasons for having a saturated inner filling solution and separating it from the external test solution. First, evaporation of the solvent will not affect the concentration of the solution and hence the electrode potential. Second, a high concentration of reactants allows for higher and stable exchange current density, which allows the reference electrode to maintain its potential when current is flowing through it. Third, any minute fluctuation in the concentration of a saturated solution will not change the electrochemical potential (and hence, the electrode potential) greatly because of the logarithmic dependence of the chemical potential on the concentration. Finally, the larger portion of the electrode potential occurs due to the large net dipole at the metal-electrolyte interface. Since the composition of the test solution is application-dependent, it would be desirable to have the reference electrode potential as independent as possible from the test solution. Liquid junction potentials are very small and will not change much by changing the test solution. However, if the test solution were in direct contact with the electrode, then the surface potential could vary greatly.
4. The interface between the test liquid and the saturated solution must be designed to minimize any form of rapid convective mixing. Such rapid mixing contaminates the reference electrode’s environment and the test solution, possibly affecting the chemical and biological reactions of interest in the test solution and definitely changing the reference potential.
5. The liquid junction potential should be the minimum possible, and should be constant with time. This is generally done by ensuring that the solvent is the same to avoid net dipole moments at this surface. Detailed liquid junction analysis has been cited extensively in literature [16,17]. Generally, electrochemical equilibrium is reached rapidly while the effects of convective mixing are hindered by the porous membrane [6]. However, if other species are allowed to interact and pass through this membrane, they can take place in the equilibrium process and the final potential can be disturbed. Equilibrium might never be reached and this process can assist in rapid leakage of the reference electrode solution.
6. The reaction should be simple enough that the exchange current density law (Equation 2) is valid. Many electrode reactions are not modeled by the Butler-Volmer equation and require many different steps for the reaction to occur. For the electrode reactions in which intermediate processes do not occur, there might be a need for the adsorption of reactant ions onto the electrode’s surface, and the energetics of the reaction become complicated. In such reactions, the equivalent exchange current density becomes quite small, possibly needing a very long time to reach equilibrium and hence are not reversible.
  The junction should be free of other possible contaminating species that can affect the reference electrode potential by changing the chemical potential of the reactants, or worse, by participating in the electrochemical reaction and making it impossible to attain equilibrium.
7. The characteristics of the reference electrode must be reproducible. Many different electrodes might have excellent stability and low drift. However, due to the limited control over the fabrication procedure, they might end up having different potentials.

Reference electrodes are expected to maintain constant electrode potentials throughout their operational lifetime, but many different processes can compromise proper reference electrode potential, and numerous studies have been performed towards minimizing these effects. In the case of the Ag/AgCl electrode, dissolution of AgCl changes the activity of the ions in the electrolyte, which affects the potential. This problem becomes much worse if the volume of the filling solution is small. Additionally, as the electrode operates, there is continuous dissolution of the AgCl layer, and once it is used up, the electrode potential changes dramatically, since AgCl is no longer available for cathodic operation. Another problem is that the filling solution can leak through the porous glass separator and cause degradation in the electrode potential due to its effects on the activity in the saturated solution and the effects on the potential of the liquid junction. Under normal conditions, the liquid junction is in electrochemical equilibrium with respect to one ion, but is not in material equilibrium. Material equilibrium takes place slowly and causes slow degradation of the reference electrode potential over time. Finally, test solution pH fluctuations may influence a shift in electrode potential and hence must be regulated accordingly [18]. When a reference electrode lacks a filling solution and is in direct contact with the test electrolyte solution, it is termed a “quasi”- reference electrode; such electrodes eliminate the liquid junction potential but do not provide stable potentials and the potentials vary significantly with the test solution. The SHE or Calomel electrodes are also viable options for miniaturization, however they are less favorable due to the intricacy of the design (requiring controlled H_2_ pressure) and incorporation of toxic mercury, respectively.

**Figure 3.**
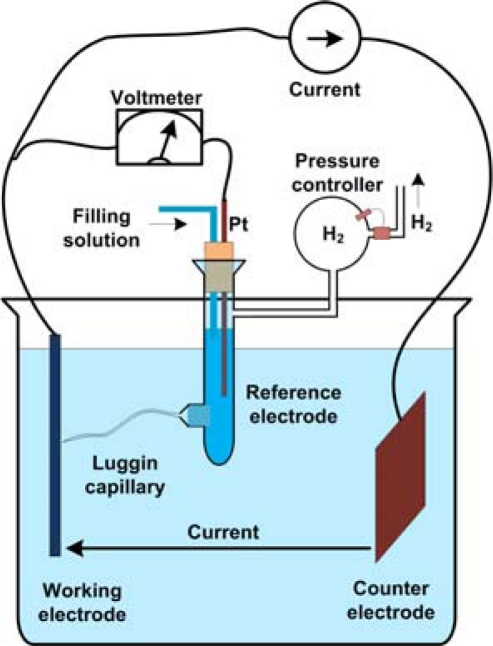
Three electrode setup with a standard hydrogen reference electrode.

A drifting electrode potential has many undesired effects. Electrochemical studies of electrode potentials normally utilize the three-electrode configuration. In this setup, shown in Figure 3, the electrode whose potential is to be measured is named the working electrode. Current is supplied to the working electrode from another electrode called the counter electrode. The counter electrode potential changes depending on the amount of current injected and hence cannot be used to measure the electrode potential. A third electrode (reference) is introduced into the solution and the working electrode’s potential is read with respect to the reference electrode, when a constant current is applied. Alternatively, a constant voltage can be applied between the working and reference electrodes and the current is read. The main advantage of the reference electrode over the counter electrode is that the reference electrode’s potential does not fluctuate, and no current passes through it in this experiment.

## 3. Common Reference Electrode Types

Depending on the application and the availability of specific ionic species in the test solution, different electrode-electrolyte systems can be used as reference electrodes. In general, a single reference electrode will not satisfy all of the requirements of an ideal reference electrode, with the result that what serves as a good reference electrode for a certain environment might be unsuitable for some other environment or application. With these considerations taken, there are a few electrode types that appear more often than others as reference electrodes in many different applications. This section briefly describes these electrodes.

### 3.1. Ag/AgCl Electrode

The silver/silver-chloride electrode is by far the most common type of electrode used in research and industry due to its simple construction. The macroscopic model is comprised of silver wire coated with silver chloride, immersed in a Cl^−^ ion rich solution such as KCl, all of which is enclosed in a glass tube and separated from the test solution via a membrane as well as a salt bridge. In such electrodes, AgCl dissolution and internal filling solution effusion pose serious threats to reference electrode stability and accuracy. The following reaction is used to characterize the Ag/AgCl electrode:

(4) $$\mathrm{AgCl}(s)+e^{-}↔\mathrm{Ag}(s)+{\mathrm{Cl}}^{-}(\mathrm{aq})$$

According to this reaction, it is expected that the chlorine ion acts as the chemical species in the operation of the electrode, and its activity, which is related to its concentration, has a significant impact on the electrode potential [19]. The electrode potential can be obtained from the Nernst equation (Equation 1) by substituting appropriate quantities from equation 4 and is:

(5) $$E_{\mathrm{Ag},\mathrm{AgCl},{\mathrm{Cl}}^{-}}=E_{\mathrm{Ag},{\mathrm{Ag}}^{+}}^{0}+\frac{\mathrm{RT}}{F}\text{ln}\;K_{s}-\frac{\mathrm{RT}}{F}\text{ln}\;a_{{\mathrm{Cl}}^{-}}$$

where *K_s_* = *a*_*Ag*^+^_ · *a_Cl_*_^−^_ is the activity solubility product of silver chloride and is a constant which is usually incorporated into the standard potential of the silver/silver chloride electrode to produce:

(6) $$E_{\mathrm{Ag},\mathrm{AgCl},{\mathrm{Cl}}^{-}}=E_{\mathrm{Ag},{\mathrm{Ag}}^{+},{\mathrm{Cl}}^{-}}^{0}-\frac{\mathrm{RT}}{F}\text{ln}\;a_{{\mathrm{Cl}}^{-}}$$

In a rich chloride filling solution, the flow of current which causes a change in chloride content, will not affect its chemical potential and is the reason for this electrode’s stability. The standard electrode potential for the Ag/AgCl electrode is 0.22 Volts at unit Cl^−^ activity. As the Ag/AgCl electrode is often used with saturated HCl, the practically used electrode potential is 0.199V. The exchange current density for silver electrodes in 1M perchlorate (XClO_4_) solution is around 1A/cm^2^ [14].

### 3.2. Calomel Electrode

The Saturated Calomel Electrode (SCE) consists of mercury, which is covered by a mercury chloride paste (Hg_2_Cl_2_ calomel), all of which is in contact with a saturated KCl solution, enclosed in glass tubing; a platinum wire is used for external contact [20]. The benefits of such an electrode are ease of construction and stable potential; however, working with mercury is hazardous due to its toxicity. The associated equation for the redox reaction of the calomel electrode is as follows:

(7) $${\mathrm{Hg}}_{2}{\mathrm{Cl}}_{2}(s)+2e^{-}↔2\mathrm{Hg}(l)+2{\mathrm{Cl}}^{-}(\mathrm{aq})$$

The Nernst relationship between the potential and the composition is:

(8) $$E_{\mathrm{cal}}=E_{\mathrm{Hg},{\mathrm{Hg}}_{2}{}^{2+}}^{0}+\frac{\mathrm{RT}}{2F}\text{ln}\;K_{s}-\frac{\mathrm{RT}}{F}\text{ln}\;a_{{\mathrm{Cl}}^{-}}$$

where $K_{s}=a_{{\mathrm{Hg}}_{2}^{2+}}\cdot a_{{\mathrm{Cl}}^{-}}^{2}$ is the activity solubility product of mercurous salt chloride and is a constant which is usually incorporated into the standard potential of the silver/silver chloride electrode to produce:

(9) $$E_{\mathrm{cal}}=E_{{\mathrm{Hg}}_{2}{\mathrm{Cl}}_{2}}^{0}-\frac{\mathrm{RT}}{F}\text{ln}\;a_{{\mathrm{Cl}}^{-}}$$

Evidently, the potential depends upon the chlorine ion according to the Nernst equation, so maintaining KCl saturation is vital for a stable potential. The standard electrode potential for the saturated calomel electrode is 0.245 volts. The exchange current density for the Calomel electrode is approximately 1A/cm^2^ [21].

Although miniaturized mercury-containing electrodes have been manufactured [22–23], they often require some form of gel matrix to keep the mercury electrode in place. Incorporation of such gels is not readily compatible with microfabrication techniques. The availability of simpler Ag/AgCl electrodes with high stability, and also the toxicity of liquid mercury, makes the SCE not a very attractive candidate for miniaturization.

### 3.3. Hydrogen Electrode

The **s**tandard **h**ydrogen **e**lectrode (SHE) is an important electrode because its standard potential is set at 0, and all other electrodes’ standard potentials are measured with respect to it. The construction involves a platinum surface covered with black platinum. The black platinum helps facilitate reduction of protons and acts as a catalyst for the electrode reaction. In addition, a coated platinum surface has a much larger area than bare platinum, which enhances the exchange current density. The platinum is immersed in an acidic solution, and the reaction progress can be seen as hydrogen gas bubbles over the platinum surface. The entire cell is contained within a glass enclosure and a pressure controller keeps the pressure of hydrogen fixed to maintain the electrode’s potential. The associated reaction is as follows:

(10) $$2H^{+}(\mathrm{aq})+2e^{-}↔H_{2}(g)$$

The Nernst equation for this half cell is $E=E_{0}+\left(\mathrm{RT}/2F\right)\text{ln}\left\{{\left(a_{H^{+}}\right)}^{2}/P_{H_{2}}\right\}$ and suggests that the hydrogen ion activity is predominantly responsible for the observed potential in this case. The absolute electrode potential of the SHE is estimated to be about 4.44 volts at standard conditions of 20°C temperature and 101.325 kPa pressure [13,24]. The exchange current density for hydrogen electrodes has enjoyed a great research interest (see, for example, Reference [25] and the references therein), possibly because of the importance of the hydrogen electrode as a universal reference, the simplicity of characterization of these electrodes, and the significance of the hydrogen evolution reaction in the study of acids, bases, buffer solutions, and many biochemical processes. Several values of the exchange current density have been measured for the hydrogen evolution reaction in the literature, and many comprehensive tables are documented for the exchange current density with different electrolytes, activities, and different electrode materials. The interested reader is referred to [26,27] for very good studies of the different values of the exchange current density.

Although deposition of platinum electrodes is readily available in microfabrication, designing a completely microfabricated SHE can prove to be a hard task. Aside from needing proper gas chambers for the hydrogen gas (which might not be semiconductor-friendly), there is a need for microfabricated pressure regulators that would keep the hydrogen pressure constant, since the gas pressure affects the electrode’s potential.

### 3.4. Other Pseudo-Reference Electrodes

There are several electrochemical systems that are used as pseudo reference electrodes. For example, using Ag/AgCl without the inner filling solution would serve as a pseudo reference electrode where the potential of the electrode is constant in a defined analyte that does not vary with time. An important pseudo reference electrode system is one that uses iridium/iridium oxide. Typically, the set-up consists of an iridium metal which is then modified with an oxidizing agent to form an oxide coating. The major problem associated with this electrode is its sensitivity to hydrogen ions. In other words, the test solution pH determines the operating potential of this reference electrode, and hence its predominant use has been in pH sensors. The associated equations for this electrode are dependent on its application. For example, in iridium oxide deposition processes [28], the two following equations describe the electrode reaction:

(11) $$\begin{array}{l} {\mathrm{IrO}}_{2}+H^{+}+e^{-}↔IrOOH\\ IrO{\left(\mathrm{OH}\right)}_{2}+H^{+}+e^{-}↔Ir{\left(\mathrm{OH}\right)}_{3} \end{array}$$

It can be seen that these reactions are expected to be pH sensitive. Other electrode reaction equations are possible in which the anions of acids (X^−^ in HX acid) and cations of bases (M^+^ in MOH) play a significant role [18]. The uncertainty of the exact electrode reaction and the strong pH dependence result in an uncertainty in the electrode potential, and this renders the iridium oxide electrode unsuitable as a reference electrode in situations where the pH value is varying and it is seldom used as a true reference electrode. However, when used as a quasi-reference electrode with a phosphate buffered saline (PBS) solution at pH 7, the standard electrode potential is around 0.52 volts [30,31]. Therefore, in medical and implantable applications where the pH is regulated, its bio-compatible properties, mechanical stability, and high exchange current density, sometimes allows it to function as a suitable reference electrode [29]. The exchange current density for IrO_x_ electrode in 1M perchloric acid is generally in the range of 0.1–1 mA/cm^2^, although there is reported deviation of these values and a strong dependence on the surface geometry and charge on the electrode [37].

In addition to the aforementioned reference electrodes, many other reference electrodes are less frequently used. The mercury-mercury oxide and mercury-mercurous sulfate electrodes are used when it is not desirable to have chloride ions. The standard potentials of these electrodes versus SHE in 1 M of NaOH and 0.5 M of H_2_SO_4_, are 0.14 V and 0.68 V, respectively. The copper-copper sulfate electrode, with an electrode potential of 0.316 V in saturated CuSO_4_, is the reference electrode most commonly used for characterizing other electrodes for industrial cathodic protection. Similarly, hydrogen reduction on noble metal such as palladium has also been used as a quasi reference electrode [32]. The palladium hydride electrode takes advantage of the fact that palladium metal is capable of storing 900 times its volume of hydrogen in its lattice due to the formation of α + β Pd hydride phase. This phase is known to behave as a hydrogen electrode and therefore yield a Nernstian dependence of potential on pH. The potential of this phase is independent of the hydride composition (its hydrogen loading) and holds a steady value of +0.050 V versus RHE. Thus, electrolytically generated hydrogen can be stored in the metal, thereby maintaining its potential till the time that there has been a significant efflux of hydrogen from the lattice into the environment. This provides a stable potential at the electrode when the pH of the solution is stable. Exchange current densities of 133 A/m^2^ have been reported for this electrode [33].

## 4. Miniaturization and Fabrication Techniques

Miniaturization and microfabrication of reference electrodes is an important prerequisite to realizing integrated microsensors and lab-on-chip devices. Many problems are faced during miniaturization, most notably the rapid dissolution of small electrode volumes such as Ag/AgCl and correspondingly short lifetime of the electrodes. Thinner electrode layers get depleted faster and the underlying support lead gets exposed to the solution, creating mixed potentials. Therefore, most attempts at microfabrication use some form of diffusion barriers to limit the dissolution of the electrode. The main areas of improving microfabricated reference electrodes are in the areas of electrode deposition and exposure to the solution, electrode activation, or the design and control of the diffusion barrier.

**Figure 4.**
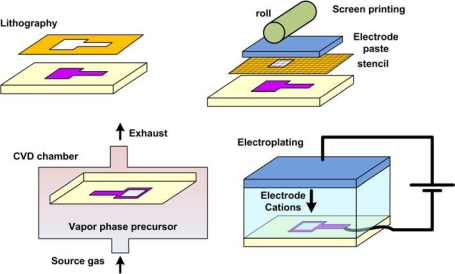
Different methods of micro-deposition of electrode’s material.

Fabricating the reference electrode is generally done in one of the following ways (Figure 4):

1. **This film deposition:** Evaporation, sputter deposition or chemical vapor deposition (CVD) of the electrode material (e.g., Ag) onto a lithographically patterned metallic lead.
2. **Electroplating:** The substrate is electrochemically treated in a bath containing cations of the required metal. Electroplating allows for higher thicknesses of the electrode material [34].
3. **Screen printing:** This involves pressing the electrode metal “ink” onto a surface using a blocking meshed stencil. This technique can produce the highest thickness of electrode material [35], but is normally done as a manual step and not integrated with automated processing techniques.

After the base metal is deposited, an activation step is generally needed to create the reference material. In the case of Ag/AgCl, the silver layer must be chlorinated to produce the coating AgCl. For calomel electrodes, a calomel layer Hg_2_Cl_2_ must be introduced. Standard hydrogen platinum electrodes are generally coated with platinum black to enhance charge transfer dynamics. The modification technique used depends on the type of chemistry needed and on the resulting surface geometry. Activation is usually done by one of the following techniques:

1. **Direct mixture deposition:** As in the case of screen printing Ag/AgCl, a compound paste is deposited directly onto the metallic surface. However, the adhesive properties of this layer to the supporting electrode might not be very good, and reliability of the electrode will depend on the homogeneity of the paste used.
2. **Ion exchange reaction:** A spontaneous chemical reaction can form the needed film. In the case of Ag/AgCl, bathing the electrode in a solution of FeCl_3_ is a known method to form the silver chloride layer.
3. **Electrochemical coating:** An electrochemically induced reaction allows controlled surface chemistry and a reportedly more Nernstian behavior [34], albeit slower than ion-exchange reactions. The AgCl coatings can be electrochemically made by anodization of silver in a HCl bath. Platinization of platinum electrodes is also done using an electrochemical coating reaction.

**Figure 5.**
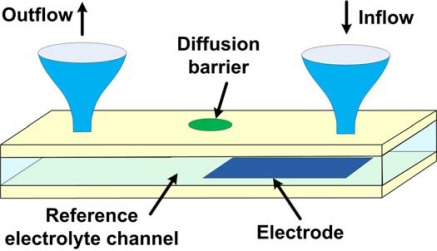
Illustration of a flow-through channel.

The final stage in designing true reference electrodes (containing liquid reference solution) is the design of the reference solution chamber and the interface to the test solution. The reservoir is generally designed as a flow-through microfluidic channel (Figure 5), allowing replenishment of the reference solution. The diffusion barrier can be constructed in many different ways, some of which are listed below.

1. **Porous materials:** Introduction of naturally porous materials, such as agarose gel, between the reference and test solutions.
2. **Gels:** Use of synthetic gels, such as polyacrylamide gel.
3. **Porous glass:** This is most used in conventional reference electrodes.
4. **Heterogeneous designs:** Specially engineered multilayer structures can provide different reaction chemistries at the two different solution interfaces, providing selective passage of potential-determining ions.
5. **Nano-porous membranes:** Nano-channels are made on polymer membranes using excimer laser ablation. The size of the nanopores can be adjusted to achieve the required junction resistance. Such membranes were used for conventional reference electrodes [36] and are seen as good candidates for the microfabrication of true reference electrodes.

Much of the work in previous years has attempted to synthesize miniaturized (or microfabricated) reference electrodes in one form or another. The following sections review some of the major accomplishments in different reference electrode designs.

### 4.1. Micro Ag/AgCl Electrodes in Capillaries

Initial attempts at miniaturization were to use a fine capillary tip in which the Ag/AgCl wire was submerged in an electrolyte solution. This derives from the Haber-Luggin capillary (as depicted in Figure 4) that has been used since the 1950's to measure the potential of the solution very close to the working electrode. For example, the tip of the capillary has been coated with polyacrylamide gel that is used as a salt bridge with low electrolyte flow rate [38]. The gel is made conductive by incorporating 3M KCl during polymerization, which also ensures that the junction potential is low. In another work [39], a 2 ml glass syringe was used as the reservoir for a thin Ag/AgCl reference element. The thin capillary tip was filled with a salt bridge and was able to access small volumes of fluid in a restricted area. The stability and short term reproducibility of this electrode was found to be comparable with laboratory-based macro-scale reference electrodes. In another innovation, a micropipette puller was used to pull a 1 mm internal diameter, pH sensitive glass capillary, and to produce a capillary tip as small as 1–2 μm [40]. The tip was capped using an agar gel saturated with KCl. An Ag/AgCl wire of 0.13 mm was placed inside the capillary which was filled with a variety of reference solutions. This configuration, combined with non-aqueous inner filling solution, was suggested as a stable pH sensor for biological studies such as measurements of pH values in the proximal and distal tubules of the kidneys of rats and dogs. A similar configuration has been used for measurement of potential in confined spaces such as living cells [41]. In another variation, silver was vapor deposited on the outside of a pulled glass capillary with 10 μm outer diameter, part of it was converted to AgCl electrochemically and used as a reference electrode for neurotransmitter measurements in extremely small volumes [42]. Similarly, disposable polypropylene pipette tips stuffed with high density polyethylene microporous membrane at the tip, filled with saturated KCl with Ag/AgCl wire inserted have also been demonstrated [43]. These kinds of miniaturized electrodes have also been assembled inside 23–26 gauge hypodermic needles as dip and spear type probes for routine pH measurements in microdialysis and clinical pH monitoring [44].

### 4.2. Microfabricated Solid-State Ag/AgCl

**Figure 6.**
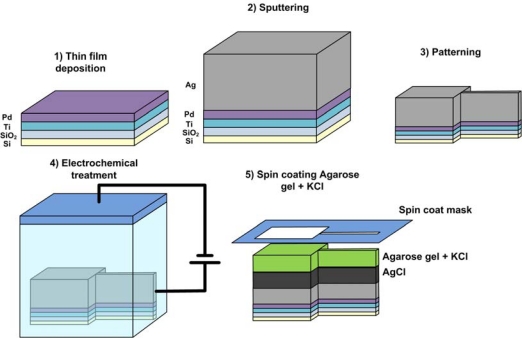
Steps in microfabricating a fully solid-state reference electrode [48].

As previously discussed, the Ag/AgCl is notably the most practical and effective electrode to use in research as its macroscopic version is simplest and safest to manufacture. Also, the kinetics of cholride ion adsorption and the mechanism of silver chloride layer formation have been studied and electrochemically optimized in great detail [45,46]. Due to its popularity in the macroscale, Ag/AgCl reference electrode is most often targeted for miniaturization and implementation in various bio-sensors. Particular attention has been given to solid-state reference electrodes in favour of eliminating the liquid junction and associated potential present in reference electrodes employing the filling solution. Details of such electrode operation and associated chemistry have been documented [47]. Typically, the filling solution is replaced with a solid-state exchange membrane doped with the ions required for the electrode equilibirium. Having solid-state support limits or eliminates convective mixing-induced drifts. One way to accomplish a solid-state support is using electrolyte-infused gels. For example, agar gel saturated with KCl has been spin coated [48] and screen printed [49] on microfabricated Ag/AgCl thin films (Figure 6). This modification has a significant effect in making the solid state reference electrode insensitive to pH in the range of 4–10 [44] and Cl- ion concentration in the range of 10^−6^ M to 0.3 M [45].

In another study, 1-dodecyl-3-methylimidazolium chloride, an ionic liquid, was incorporated into a **p**oly**v**inyl **c**hloride (PVC) matrix which was coated over the silver/silver chloride electrode [50]. The ionic liquid replaced KCl in the solid matrix and maintained constant concentration of chloride ions in the internal solid electrolyte, providing a constant potential of reference microelectrodes in solutions of varying concentration of Cl−, NO_3_− and Br− anions. Ionic liquids have low vapor pressure and volatility, and hence do not have drying problems associated with KCl saturated gel electrodes when stored for long durations after manufacture [50].

**Figure 7.**
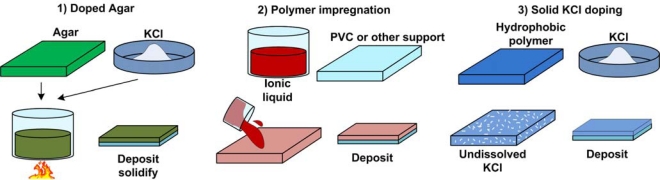
Different methods for synthesis of a solid-state membrane [48–58].

In other studies **p**oly**v**inyl **c**hloride (PVC) [51–53] and epoxy coatings [54] have been used to protect bare Ag/AgCl electrodes from dissolution in the analyte solution while polyurethane, nafion [55,56], and silicone rubber coatings [57], have been used as membrane material in order to prevent the dilution of KCl in the agar gel layer and enhance the electrode’s stability. The polymer used depends on the application and susceptibility to degradation, and the most commonly used ones, polyvinyl chloride, polyurethane, or nafion, were shown to have poor reproducibility when integrated in solid-state reference electrodes. In another solid state construction, KCl powder was added to a UV curable hydrophobic dielectric polymer (Dupont 5018) and screen printed on top of Ag/AgCl layer [58]. The KCl powder was able to dissolve in water forming KCl saturated microchannels when dipped in the analyte solution. Since the KCl was in the powder form, this type was preparation was sutiable for long term storage of the electrode. Figure 7 illustrates some of the techniques used for solid-state electrode fabrication.

### 4.3. Microfabricated Ag/AgCl with Filling Solution

**Figure 8.**
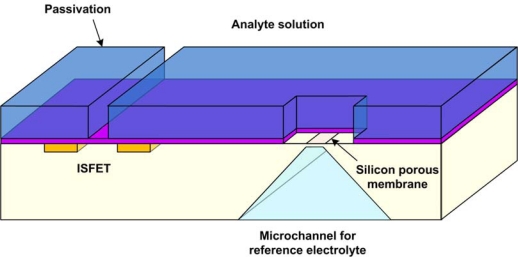
Bulk micromachined reference electrode channel and membrane [59,60].

Although the solid state reference electrodes are more suitable for construction and integration, they do have problems associated with stability due to the finite amount of the KCl present and in the variation of Cl- concentration due to efflux or influx from the electrode matrix. The reduced mobility of Cl- through the membrane can also limit the exchange current density and introduce capacitive effects within the membrane. For these reasons, designing old-fashioned reference electrodes at the microscale is still very attractive. Several implementations of reference electrodes with special microfabricated reservoirs and separation membranes have been investigated to achieve true Ag/AgCl micro reference electrodes. For example, a miniature liquid junction microfabricated reference electrode has been incorporated into an **i**on **s**ensitive **f**ield-**e**ffect **t**ransistor (ISFET) based chemical sensing system [59,60]. The reference electrode was integrated with the ISFET by etching a cavity from the back side of the silicon substrate on which the ISFET has been previously fabricated such that a thin porous silicon membrane remains on the surface (Figure 8). This serves as the membrane separating the inner and the outer solutions. The cavity was filled with the reference solution and a Ag/AgCl wire was inserted into it. Other researchers have used thin planar porous glass [61], a hydrogel layer [62] or a membrane with a pinhole junction [63] to separate the inner and the outer solutions.

**Figure 9.**
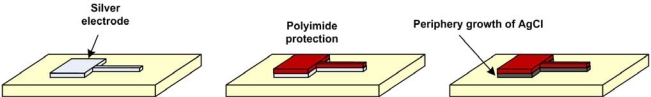
Bulk micromachined reference electrode channel and membrane [64].

One of the key problems with microfabricated Ag/AgCl reference electrodes is the depletion of Ag or AgCl and the resultant degradation in performance. As thin-film technologies are used to deposit Ag and then convert some of it to AgCl, the lifetime of microfabricated reference electrodes is low. An interesting modification has been employed by confining the thin film Ag between two insulating layers to expose minimal surface area to be in contact with the reference solution [64]. In this process, depicted in Figure 9, silver was deposited by evaporation, patterned lithographically and chemically etched to define the shape of the reference electrode. Subsequently, an insulating thin film (polyimide) was deposited and patterned in such a way that the edges of the silver layer are exposed. The AgCl layer was then electrochemically grown from the periphery of the silver pattern and exposed to the filling solution. Experimental results demonstrate higher stability with open circuit potential drift of <1mV over 24 hrs. Also, several modifications were later introduced to this design. The technique for growing the silver chloride layer was changed in favor of oxidizing the metal with FeCl_3_; an ion exchange membrane made of cellulose acetate was integrated, as well as the addition of a concentrated KCl filling solution [65]. Finally, in more recent studies [66], the electrolyte layer was made from KCl powder paste, a photo-curable polymer was used as an exchange membrane, and the AgCl layer was grown over the silver layer from a thin slit in the polyamide layer. Also, FeCl_3_ has been repeatedly cited as an effective oxidizer capable of producing uniformly thin and robust coatings suitable for reference electrode applications [34,67,68].

**Figure 10.**
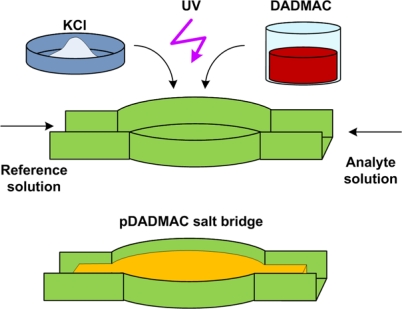
UV polymerization and chloride trapping in a salt bridge [69].

In another study [69], a planar microfluidic configuration has been adopted for miniaturization and integration of the conventional Ag/AgCl reference electrode design with a microsensor. In this work, microchannels were fabricated out of **p**oly-**d**i**m**ethyl **s**iloxane (PDMS), a commonly used material in microfluidics. The main microchannel consisted of the working (sensor) and counter electrodes. A side channel consisting of an Ag/AgCl wire inserted into its reservoir, interfaces with the main channel close to the electrodes through a salt bridge. A salt bridge is formed at the T-junction separating the main channel from the side reference channel by UV **p**olymerization of **d**i**a**llyl**d**i**m**ethyl**a**mmonium **c**hloride (pDADMAC) which functions as an ion exchange membrane (Figure 10). The electrode maintained a potential range of 15–18mV with respect to a commercial Ag/AgCl for around 30 hrs.

In other instances, screen printing techniques have been used to create the reference electrode [70,71]. Silicon was used as the substrate, and it was patterned with Ti and later with Pt for adhesion between the layers. Following this, Ag/AgCl was screen printed on the platinum and appropriate post process curing and baking procedures were carried out. Techniques such as pulse layer deposition may be used to deposit various layers, and different deposition techniques lead to different grain sizes. It was found [71] that thicker grain size is optimal for the operation of Ag/AgCl electrodes.

Other, more irregular membrane materials such as polytetrafluoroethylene have been suggested in light of their easy integration and pore size control [72]. In many studies, much attention is given to the behaviour of the filling solution within the electrode. It has been noted that higher concentrations of filling solution ions result in a more rapid dissolution of the AgCl layer [68]. As well, forcing hydrophobic and hydrophilic characteristics onto various surfaces of the glass capillary may allow for tight control over filling solution effusion [73]. The possibility of using miniaturized quasi-reference electrodes also arises; however this will cause dissolution of the AgCl layer. Attempts have been made to use Ag/AgI [74] and Ag/Ag_2_S [75] interfaces, due to a lower solubility constant.

In all cases of miniaturization of reference electrodes dealing with a filling solution, an issue arises related to its evaporation over time, leading to loss of accuracy in the potential. To circumvent this problem, it is possible to use a liquid ion-exchanger alternative to KCl [50,76,77]. The ions present in the liquid will maintain their concentration due to the constant volume contraint as evaporation of water is not possible. Coupled with an ion exchange membrane, this scheme may be vey effective at maintaining constant potential.

### 4.4. Microfabricated Ag/AgCl without Filling Solution

As in the case of solid-state reference electrodes, quasi-reference electrodes are becoming very popular in microsystems due to their simplicity of manufacture and relatively good performance during their intended lifetime. Quasi-reference electrodes do not have an internal electrolyte, and their potential can therefore be severely affected with variations in the test solution. However, the absence of a separating membrane makes the design much simpler, and allows lower drifts due to convective mixing. Furthermore, for many applications, especially biosensors, integrated electronic circuits can be built to compensate or cancel the effects of reference electrode drift.

**Figure 11.**
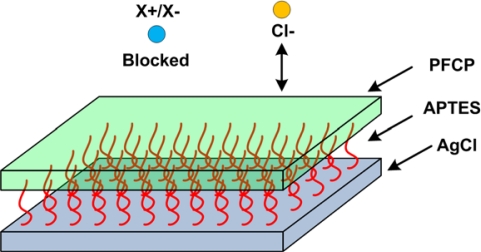
Blocking cross-contamination with self-assembled molecules [78].

Quasi-reference electrodes suffer from the rapid change in potential with contaminating species, an effect that is avoided in regular reference electrodes by employing highly concentrated filling solutions. To minimize the effects of cross-contamination, a microfabricated Ag/AgCl electrode was coated with an adhesive **a**mino**p**ropyl**t**ri**e**thoxy**s**ilane (APTES) layer, followed by a layer of **p**er**f**luoro**c**arbon **p**olymer (PFCP) to block contaminating ions (Figure 11) [78]. Although the electrode showed severe shifts (expected) with Cl^−^ ion concentration change, it seemed to be immune to other halide contaminants and pH of the solution. It also showed excellent long-term stability with less than 10mV of drift in over 80 days.

Another example of a quasi-reference Ag/AgCl electrode is given in [34], where the silver layer was deposited by electroplating it on thin film microelectrodes patterned by conventional lithographic techniques. A 5 μm thick silver layer was deposited and the silver chloride layer was subsequently grown on it by chemical treating the electrode in FeCl_3_ solution for 50 s. It was found that the electroplated electrodes showed a drift of less than 2mV for at least 1000 min before failure, while the electrodes without electroplated silver showed large potential variations within a few minutes of measurement.

**Figure 12.**
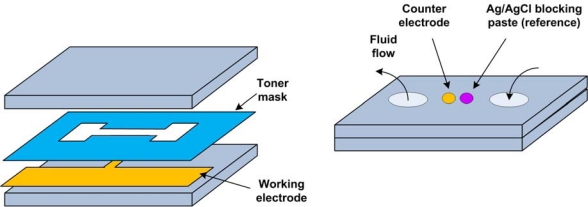
A microchannel made using toner mask, with the reference Ag/AgCl introduced as a paste blocking drilled holes [79,80].

An attempt at cost-effective production of three-electrode microstructures, including a quasi-reference electrode, was recently presented in [79], and subsequently in [80]. The design is illustrated in Figure 12. A microchannel was created using a laserjet-printed cavity sandwiched between two polycarbonate films, so that the thickness of the toner determined the thickness of the microchannel. The top film was drilled to introduce applicator holes such that a through-cell design was achieved. Two drilled holes were filled with graphite and silver composites and contacted with metallic leads from the outside, forming the counter and reference electrodes, respectively. Although this and similar quasi-reference electrodes will not have high stability, they are especially useful for disposable sensor applications and for rapid analysis systems where the experiment’s lifetime is shorter than the reference electrode’s drift characteristic times.

### 4.5. Miniaturized Mercury and Calomel Electrodes

Miniaturized mercury electrodes have been made inside glass capillaries [22]. These mercurous electrodes are similar to the calomel reference in that they employ mercury-based salts at the mercury interface. Mercury is electroplated onto a gold wire to form an amalgam and serve as a solid state mercury reservoir. Mercurous salt is formed on its surface by electrodeposition. The wire electrode is then immersed into an agar gel matrix, saturated with a common ion to that of the salt. The entire content is contained in a glass capillary that provides structural support for the electrode, and prevents diffusion of the inner electrolyte to the test solution. This is critical when conducting measurements *in-vivo*, as the inner electrolyte is hypertonic and can damage tissues [20]. The conventional calomel scheme has also been miniaturized [20], employing mercury on top of a calomel/cotton mix, all encapsulated in a glass capillary. A conventional glass capillary (3 mm i.d. and 4 mm o.d.) was pulled using a pipette puller to obtain a tip of 300–400 μm. The tip was filled with 10% NaCl solution in 2% agar and allowed to solidify. Next, a cotton or paper matrix saturated with calomel was dipped in NaCl and inserted on top of the agar layer. This is topped off with liquid mercury to form the reference electrode [20]. Similar configurations but using solid silver amalgams interfaced with mercury chloride paste has also been reported [81]. Finally, augmentation to a capillary based miniaturized design has been made in calomel electrodes by flowing the inner saturated KCl solution at a rate of 1–2 μL/hr through the capillary into the sample fluid to replenish liquids at the liquid junction. This method provided a much more stable liquid junction potential [23] and hence increased the reproducibility of measurements to less than 1mV. Furthermore, the stabilization of the resting potential (to within 10.1 mV) was achieved at 1–3 minutes, as opposed to 3–15 minutes for standard reference electrodes.

### 4.6. Miniaturized Hydrogen Electrodes

Miniaturization of the hydrogen-platinum reference electrode has been by far the most complicated. This is because a miniature hydrogen gas source is required to provide saturation hydrogen concentration over the platinum electrode surface in order to maintain its potential. As such, numerous methods have been devised to eliminate the hydrogen source and use ion doped membranes to eliminate the need for an acidic solution. A particularly pertinent effort to fabricate a miniature hydrogen electrode is presented in [82]. This involves a very effective design which entails using perfluorosulfonic acid polymer tubing (0.3 mm inner diameter and 0.6 mm outer diameter), which is injected with a platinum black powder and 5% Nafion paste mix, and contacts a Pt wire for external contact. Pure hydrogen gas is bubbled through this tubing at 1 atm pressure from an external source. The design is attractive from a practical standpoint as it eliminates liquids and associated junction potentials. The potential of this electrode configuration is stable for acidic and neutral test solutions, but varies significantly in alkaline solutions. The electrode has high stability (<0.2 mV variation over 1 hr) against the SHE in H_2_SO_4_ solution.

In another interesting study [83], that is more suited to miniaturization, a Pt wire was inserted into a sealed glass capillary that was filled with an acidic electrolyte under vaccum. H_2_ gas was electrolytically generated and forms a bubble at the top of the sealed capillary. Due to capillary action, there is a thin film of electrolyte on the Pt wire that is thin enough to allow rapid diffusion of H_2_ gas from the bubble to the electrolyte to maintain its concentration. Stable potentials with less than 0.5 mV deviation from SHE have been obtained over a period of several days [83]. The lifetime of this electrode depends on the permeation of the H_2_ gas from the bubble to the outside environment, and this was calculated to be 5 years in this configuration. A variation of this method by filling a tube with hydrogen and sealing it has also been demostrated [84]. Another approach to miniaturization used a dynamic reference hydrogen electrode [85,86]. In this configuration [86], a constant current of 1 mA/cm^2^ is passed between two pieces of platinized platinum electrode placed close to each other in a acid or alkaline medium, leading to generation of H_2_ gas at the cathode and O_2_ at the anode. The electrodes are arranged in such a way that the cathode, which now has the configurations of a hydrogen reference electrode, is below the anode and the evolution of O_2_ gas at the anode does not affect the gaseous composition near the cathode. A stable potential with variation of less than 5 mV over 5 days was obtained using this configuration [86]. The same configuration has been microfabricated and a protective polymeric membrane of poly hydroxyethylmethacrylate, a hydrogel, has been added to provide potential stability to varying flows [85].

Palladium hydride electrodes [32] that operate on the same electrochemical reaction have also been miniaturized [33]. Here platinum microwires were sealed in glass pippetes to form microelectrodes on which 25 μm Pd was electrodeposited from a 40 mM (NH_4_)_2_PdCl_4_ plating bath. Surfactants were used to create a nanoporous deposit that increases the surface area and provides more stability. The nanostructured electrode was biased at −0.2V to generate hydrogen that gets absorbed by the Pd. The completion of hydrogen loading is indicated by a plateau of the current at the applied potential. The stability of these loaded films depends on the phase present. For example, β films with H/Pd of 0.59 and 0.51, respectively, took 15 and 10 min to stabilize. However, α+β films with H/Pd between 0.42 and 0.28 reached a stable potential almost instantaneously, while the potential of an α film with a H/Pd of 0.1 rose continuously [33]. The phase composition can be deduced by measuring the resistance of the material, as has been shown in [32]. Stable potential of −0.71V could be maintained for 1–3 hrs in deaerated solutions, and the potential varies linearly over pH range from 2–12.

### 4.7. IrOx

Due to its high integrity (insoluble in aqueous media), low impedance, lower toxicity than AgCl, and low reactivity which make it easier to handle, iridium oxide is a promising electrode for miniaturization. Typically, fabrication methods are similar those employed in Ag/AgCl electrode fabrication. The most common method for microfabrication is sputter deposition of Ir thin film and electrochemical oxidation of a portion of it to oxide in an oxidising solution such as 0.1 M Na_2_HPO_4_ [29]. Other methods include exposure of the deposited Ir surface to a strong oxidisng melt bath such as Li_2_CO_3_ 0 and direct electrodeposition of IrO_x_ onto a substrate using a highly basic solution of salts such as Na_3_IrCl_6_ [88], IrCl_3_ [87] or IrCl_4_ [89]. These electrode show highly linear pH response where their potential decreases with the solution pH [29,31]. Potential uncertainty of less that 20 mV for 9 days has been reported for electrodeposited IrO_x_ electrodes. Reproducibility in the deposition process was also good with 4 mV standard deviation between several electrodes fabricated in a similar fashion 89. Certain fabrication methods may eliminate the use of an expensive iridium microwire; however, this may compromise the stability of the potential.

Table 1 provides a summary of many different microfabricated reference electrodes found in the literature. As shown, most attention is paid to Ag/AgCl electrodes. It is also noted that the most studied criterion of operation is the stability of the reference electrode potential with time. While this is indeed the most critical design parameter, there are many more factors that are seldom reported.

- First, current-voltage relationships (Tafel plots) of the electrodes are required to deduce the exchange current densiy, as its value differs with device parameters. A poorly designed Ag/AgCl reference electrode might not have its nominal exchange current density, and this is critical when discussing the dynamics of the electrode.
- Second, the internal filling solution is very often disregarded and the fabricated electrode is used as a quasi-electrode. This makes the reference potential very sensitive to the conducting ion activity, which can vary in the test solution. Unless the test conditions are extremely well controlled, the reference electrode’s potential will vary.
- Third, only a few publications document the effect of adding different species on the electrode’s potential. Most of the literature focus primarily on pH dependence. Potential contaminant species for the sensor in question must be identified and their effects on the reference electrode potential studied.
- Fourth, the choices for the test solutions and filling solutions vary, making the comparison of the reference electrodes’ performances complicated. A standard reference solution should be incorporated for each electrode type. This solution might be chosen to be that of highest relevance to the application using the electrode of interest.

## 5. Applications of Reference Electrodes in Chemical and Biomedical Sensing

Although some chemical sensors, such as gas sensors, could be designed using solid-state devices and utilizing work function differences [93] or capacitive sensing [94] due to gas adsorption, most chemical sensors test for chemicals in aqueous solutions. These involve pH sensors, blood glucose and oxygen sensors, ion sensors and many others. Bio-electrochemical sensors are typically used in aqueous bio-compatible media. For these sensors, the reference electrode is indispensible. There are many different sensor implementations, but they use one common principle - the potential of a sensitive electrode is changed by interaction with the species of interest. This change in the potential can be sensed in many different ways. Static methods involve applying a constant current and monitoring the voltage change of the electrode, or applying a constant voltage and monitoring the current change. On the other hand, dynamic methods of sensing are also quite common and can potentially allow a higher sensitivity to be obtained. Dynamic methods include impedance measurement, capacitance-voltage (CV) measurements, cyclic voltammetry, and noise analysis.

Currently, both macro- and micro-scale electrodes are used for biosensing applications. The advantage of macro-scale electrodes is that the problems associated with concentration differences, filling solution evaporation, thin film dissolution and liquid junction potentials, do not have a pronounced effect on the electrode potential. However, due to their bulky nature, they are harder to integrate into miniature biosensors. Miniaturized reference electrodes are more easily integrated, but they suffer from potential drift due to the aforementioned problems.

In biosensors, reference electrodes are either the dominant sensing device, or are integrated alongside semiconductor devices such as **f**ield-**e**ffect **t**ransistors (FETs). In FET systems, reference electrodes serve to fix a potential at a certain level to provide a bias for the gate of the transistor-based sensor, which is often exposed to an electrolyte solution. Such a device, named the **i**on-**s**ensitive FET (ISFET), was initially used in pH sensing due to the activity of silicon dioxide layers. In systems where electrodes intrinsically comprise the sensing device, they are used to observe a change in potential or current through an electrochemical system. Also, in some instances, electrodes are used to carry out impedance measurements. In a particular study [9], micro-electrode systems were used to characterize the surface of the outermost layer of the skin. The response of actuating and recording electrodes is very sensitive to the abundance of ions in the sensing medium. Thus, their electrical performance is characterized before actual usage. Sensor electrodes, on the other hand, are selectively sensitive to one particular ion. Examples of such devices are given in [95–98] and reviewed in [99].

### 5.1. pH Sensing

Reference electrodes are also typically integrated into pH sensors. pH is the measurement of H_3_O^+^ ion activity in solution, and a more precise definition is given in [100]. The activity of such ions is a function of the ion concentration. The value of pH is given by: *pH* = −log[*a*_*H*_3_*O*^+^_], and the pH scale is derived based on the potential difference between the platinum and reference electrode of the following cell [6]:

(12) $$Pt\left(H_{2}\right)|\text{Test}\;\text{solution}|\text{Saturated}\;\text{KCl}|\text{Reference}\;\text{electrode}$$

**Figure 13.**
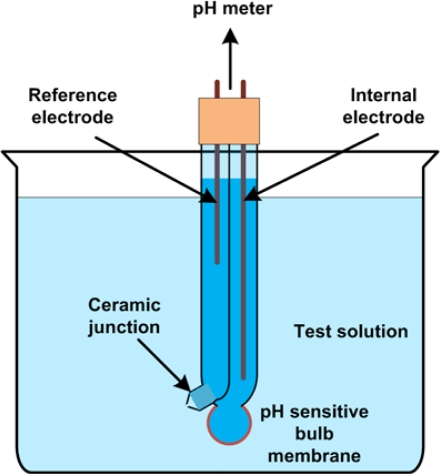
pH glass electrode in a test solution.

An apparatus constructed to measure pH is the glass electrode shown in Figure 13. Glass pH electrodes are combination electrodes, having both a reference electrode and an internal electrode. The internal electrode here is not a working electrode since it is not sensitive to H^+^ ions by itself. Both electrodes are immersed in reference solutions connected to the external solution. The junction from the reference solution to the external solution must have a very stable and predictable potential. The other junction (between the internal electrode and the external solution) is the pH sensitive glass membrane. This junction is made from a thin bulb of glass that maintains a diffusion potential difference that is proportional to the pH difference between the inner solution and the tested solution. This potential difference is a component of the total potential difference between the two electrodes and is therefore sensed when the combination is used as a galvanic cell. Since the resistance of this cell is quite high, specialized high-resistance pH meters should be used.

The operation and characterization of glass electrodes have been studied in early works [101–102]. The potential difference between the reference and internal electrode is dependent on the pH, through the glass membrane [103]. It is possible to calculate the hydronium concentration and thus pH from the measured potential using the Nernst equation:

(13) $$E=E^{\prime }+\frac{\mathrm{RT}}{F}\text{ln}\left[a_{H_{3}O^{+}}\right]$$

where *E* is the measured potential, and *E*’ is the standard electrode potential.

Miniaturization of pH electrodes for on-chip systems has been given considerable attention [31,88]. In most such pH sensors, a reference electrode is used alongside another sensitive pH electrode. Typically, iridium oxide electrodes are used due to their hydrogen sensitivity [31,88]. A stable reference electrode is needed to observe this potential change. Carbon fiber pH electrodes are manufactured by iridium oxide deposition, and the pH is tested using several distinct methods. One is the use of cyclic voltammetry in hydrogen peroxide solution, which causes oxidation and reduction reactions that influence the pH value near the substrate electrode. Another possibility is to perform a titration of a buffered solution and observe potential changes of the pH electrode.

Alternatively, pH sensors can be used to measure the chemical activities of other reactions that involve the consumption or production of hydrogen ions. An example is glucose enzyme processing using glucose oxidase, as this reaction produces hydronium species and changes the interface pH [88]. Typically, other tests such as long term pH stability are also performed [31]. There are several other pH-sensitive electrodes such as aluminum oxide, which operates on principles similar to those of iridium oxide [91]. Finally, the ISFET has been initially introduced as a pH sensor [104], utilizing the sensitivity of the exposed insulator to protons.

### 5.2. Glucose Sensors

Depending on the type of membrane used between the two electrodes in a galvanic cell, different types of monovalent cationic sensors can be constructed [105]. These are known as **i**on-**s**ensitive **e**lectrodes (ISEs). Additionally, different types of electrodes can be used to directly detect different chemical and biological substances by monitoring the effects of the chemical or biological activity on the electrode’s potential. In this case, this electrode is the working electrode. For example, modified carbon paste electrodes have been used as glucose sensors [106], and as highly selective dopamine sensors [107]. Although the sensing principle can vary amongst these working electrode sensors (e.g., electrode acting as a catalyst for a reaction, electrode reacting with sensed species, or species affecting the activity of the potential-determining ion), all such sensors consist of a sensitive working electrode and a reference electrode, with the measurement method being amperometric, potentiometric, or impedimetric.

Ion-sensitive electrodes can be used to monitor several different chemical reactions due to their specific ionic products. Different organic compounds can be sensed this way, an example of which is glucose. In [108], a glucose biosensor was made by employing a three-electrode system, with an Ag/AgCl electrode as reference and two other carbon paste electrodes modified with multi-walled carbon nanotubes. It relied on glucose modification with enzymes, and observing the impact on the electrode current. One study used Prussian Blue and Glucose Oxidase immobilized on carbon fiber nano-cones to form a glucose sensor [109] with an Ag/AgCl reference electrode. Several other glucose sensors that use the Ag/AgCl reference electrode are described in [90,110]. An alternative reference electrode which may possibly be integrated with less difficulty in a glucose sensor is IrO_x_ [1]; however, the need for a constant pH via a buffer solution such as PBS may be an inconvenience. Extensive testing analysis available for use on glucose sensors is given in [111]. One such reference electrode was implemented in a glucose sensor with a three electrode system [30]. The glucose sensing matrix consisting of electropolymerized glucose oxidase was deposited on the working electrode.

### 5.3. Gas Sensors

Another prominent application of miniaturized reference electrodes is in gas sensors such as oxygen and carbon dioxide. These systems are particularly important in biosensors which can measure physiological gas concentration, such as oxygen and carbon dioxide levels in blood. Typically, these sensors operate by sensing pH changes. Such sensors employ a reference electrode, a pH sensitive electrode, electrolyte solution and a hydrophobic permeable membrane usually made from polytetrafluoroethylene (PTFE). In one study [1], a carbon dioxide sensor was fabricated using a combination of a miniature iridium oxide electrode and an Ag/AgCl reference electrode. The pH sensitive electrode may also be replaced in favor of more suitable reference electrodes depending on operation conditions, such as the polyaniline based pH electrode [112]. The oxygen sensor operates on similar principles, but the gas sensitive membrane is particular to oxygen and the pH sensing electrode may be eliminated [113]. An integrated sensor for measuring pH and the aforementioned gases has also been reported in [114].

### 5.4. FET Based Sensors

**Figure 14.**
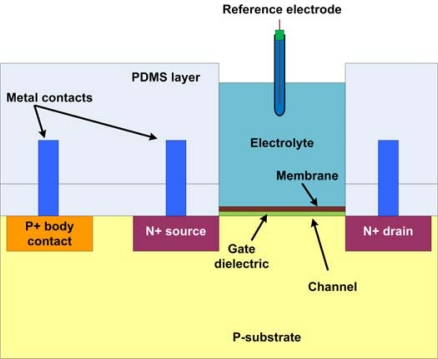
Simplified structure of an ISFET sensor.

In another type of sensor, the working electrode is made from a semiconductor, and is electrically insulated from the solution. Sensing can then be done electrostatically by measuring capacitance changes or by utilizing electrostatic transconductance effects. Examples of such a device are the **bio**logical **f**ield-**e**ffect **t**ransistor (BioFET) and **i**on-*s*ensitive **f**ield-**e**ffect **t**ransistor (ISFET), shown in Figure 14. These devices share the same features of a field-effect transistor, with doped source and drain contacts separated by a semiconductor channel of opposite doping. In a BioFET (and ISFET), the regular polysilicon gate contact which controls the amount of inversion in the channel is replaced by a conductive electrolyte solution. The insulator of the ISFET can now be coated with various different films that make it sensitive to different ions. An uncoated SiO_2_ insulator is natively sensitive to hydrogen ions and can therefore be used as a pH sensor [115]. Alternatively, oligonucleotides could be immobilized onto the ISFET and made into a DNA sensor. For this type of sensor, a reference electrode is required to set the potential of the electrolyte and to allow for channel inversion. In fact, the potential on the reference electrode determines the transistor’s operating point and has thus an important effect on the sensitivity, linearity, and noise performance of the sensor.

The real advantage to employing FETs in the possibility of miniaturization and mass production at relatively low cost, due to prospect of adopting standard **i**ntegrated **c**ircuit (IC) fabrication procedures for FET-based biosensors. Typically, the bio-sensors are extremely specific to their targets, and use antibodies or single stranded DNA (ssDNA) to bind the target molecule and induce an electrical response. Such is exactly the case associated with the BioFET; a FET device whose surface is modified with ssDNA, and relies on DNA hybridization to induce a change in electrical current response [87,116,117]. A typical BioFET is designed by removing the gate metal and replacing it with ssDNA to be immersed in an electrolyte or test solution. Also, the gate is biased by employing a reference electrode immersed in the electrolyte solution [116]. The FET is also used in protein detection such as albumin [118,119]. A study focusing on albumin detection [118] used anti-albumin to modify the gate dielectric. An Ag/AgCl macro-scale electrode immersed in an electrolyte solution was used to bias the gate using a voltage source. The device operation is based on the fact that albumin is a charged protein, and as it binds with anti-albumin, there is an accumulation of charge at the gate, which inherently induces a change in the current. There has been considerable effort to attain stability and accuracy in such sensors [120–122] dealing with modeling potential drift and preventative measures.

### 5.5. Other Types of Sensors

Other sensors are DNA-based [123] and use screen printed carbon three-electrode systems, where the working electrode is modified with double stranded DNA to allow for detection of water contaminants, or ssDNA for detection of hybridization of DNA, similar to the idea employed in the Bio-FET. An Ag/AgCl reference electrode may be used for the purpose of monitoring the reference electrode and observing changes in the electrical signal. Studies have also been targeted towards sensing hemoglobin levels in blood [124] and various amino acids and carbohydrates [125] with the aid of miniaturized reference electrodes.

An important use of reference electrodes is in medical recording, such as **e**lectro**e**ncephalo**g**raphy (EEG). The recording electrode is used to measure electrical signals that emanate from biological activities such as neuronal action potentials. The potential of the reference electrode must be constant so as to prevent contamination of the signal. When performing measurements *in-vivo*, and when dealing with implantable electrodes, the requirements for microfabricated reference electrodes become more stringent. The popular Ag/AgCl cannot be directly used without proper encapsulation since it can leak silver ions into the cerebrospinal fluid. The filling solution is taken to be one which is isotonic with the test solution (blood or spinal fluid) to maintain liquid junction stability. On the other hand, other reference electrodes that do not contaminate the environment can be used. The positive aspect is that requirements of insensitivity to pH or other cationic species can be relaxed, relying on the body’s own control systems to maintain constant salt concentrations and pH.

## 6. Conclusions and Future Perspectives

With a growing demand for the availabiliy of simple, inexpensive biosensors, the prospect of miniaturization of the reference electrode for biosensing applications becomes quite promising. A miniaturized reference electrode becomes a vital and integral part of any such biosensing device, and should be given high priority for its effective minaturization and optimization. With a range of already available fabrication techniques, and generally simple designs, as in the case of the Ag/AgCl electrode, mass fabrication of miniaturized electrodes [126–128] for biosensors [129–131] becomes a viable possibility Simplified reference electrodes have already been commercialized in the case of glucose biosensors and in sensors for blood-gas analysis. The abundance of probe analyte in the sample, relatively mild sensitivity and stability requirements and the need for low-cost sensors have enabled these devices to be the de-facto standard in these applications. It is foreseeable that the same benefits, namely: low-cost, ease of manufacture and integration of these reference electrodes will make them attractive for other biosensing applications that require higher stability and sensitivity to low concentrations of analytes in the sample. These evolving trends will need improvements in both the type, structure and fabrication of these electrodes.

Due to miniaturization, the volume and the surface area of the reference electrode decreases. This reduction in size is attractive as it enables the integration of these sensing electrodes into small devices for portable diagnositic devices. However, the penalty that one pays is a reduction in stability and lifetime. In the case of Ag/AgCl electrode, the total amount of Ag and AgCl present at the electrode determine its lifetime. Therefore, the current thin-film microfabricated reference electrodes have significantly lower lifetime of operation as compared to macroscale ones. One approach to increasing the lifetime of the electrodes will be to develop microfabrication technology to accommodate thick film Ag/AgCl electrodes, especially for applications that require monitoring of analytes over long period of time. Thick film electrodes can be fabricated using well established electroplating, screen printing and inkjet deposition methods. However, integrating them with microchannels and the rest of the sensing system proves to be a challenge and they need to be planar and have low surface roughness. Surface micromachining methods [132,133] can be used for incorporating thick film electrodes particularly with non planar topography with other elements of the sensing system.

Another approach to solving the lifetime problem is to develop methods to periodically regenerate the chemcial constituents of the reference electrode. For example, in the case of the standard hydrogen reference electrode, the H_2_ gas which is one of the components of the electrochemcial reaction that determines the potential of the electrode, can be either supplied from the outside or can be generated in-situ. The design and form factor of the electrode becomes unweildy if the it is supplied from outside. However, in-situ generation of the gas from water and storage in a matrix such as Paladium will provide a stable potential at the electrode for as long as the H_2_ gas is present and accessible to the electrochemical reaction. This method is best suited for microfabricated electrodes and electrodes of this kind will be actively investigated in the future.

The stability of the potential in a reference electrode is related to the surface area. Microfabricated electrodes have lower surface area and hence their stability is lower than the macroscale reference electrodes. This factor becomes more important in analysis that happen over long periods of time or are sensitive. One approach to increasing the stability of microfabricated reference electrodes will be to increase the effective surface area available at the surface with a fixed nominal surface area. This can be accomplished by incorporating nanostructures such as nanowires, nanosheets and nanoporous structures of the Ag and AgCl on the electrode surface. The density of the nanostrucutures increase the effective surface area and increases the stability of the microfabricated reference electrodes.

In addition to the development in Ag/AgCl reference electrodes, another active area of research in the future will be in the development of miniaturized calomel and hydrogen reference electrodes. As the microfabrication technology develops to enable complex structural fabrication increasingly possible, the reference electrode format that were difficult to accomplish in the past will be possible in the future.

In addition, macroscopic reference electrode electrochemical testing may be applied to miniaturized models, allowing for thorough characterization and optimization. The aim of such research is the eventual miniaturization of all types of reference electrodes for use in biomedical sensors, which would be readily available for bed-side use at households and hospitals without requiring much training or expertise. Inevitably, this will reduce costs and spark a new commercial industry for miniaturized biosensor development.

## Figures and Tables

**Table 1.** Summary of the performance of microfabricated reference electrodes in the literature.

**Electrode type**	**Dimensions**	**Fabrication**	**Filling solution**	**Test solution**	**Electrode potential (V)**	**Stability**	**Year**	**[Ref.]**
Ag/AgCl	13mm × 1.5mm × 0.4mm	Photolithography, Sputtering, Electrochemical oxidation	None (quasi-RE)	0.1 M KCl in 20mM NaOH/KH_2_PO_4_ buffer, pH 7	90mV vs. commercial Ag/AgCl	< 1mV in 24h, 1mV in 8h with saturated AgCl and KCl in solution	1998	[64]
Ag/AgCl	13mm × 1.5mm × 0.9mm	Photolithography, Electrochemical oxidation, Bulk micromachining	Saturated KCl, AgCl. Pin hole liquid junction	0.1 M KCl in 20mM NaOH/KH_2_PO_4_ buffer, pH 7	7mV vs. commercial Ag/AgCl	<1mV in 3h.	1998	[65]
Ag/AgCl	13mm × 1.5mm × 0.9mm	Photolithography, Electrochemical oxidation, Screen printing	Saturated KCl, AgCl. Hydrophillic Polymer liquid junction	0.1 M KCl in 50mM NaOH/KH_2_PO_4_ buffer, pH 7	8mV vs. commercial Ag/AgCl	<2mV in 100h.	1999	[66]
Ag/AgCl	N/A (macroscopic Ag wire used)	Photo-polymerization for junction polymer	1M KCl. pDADMAC plug junction	PBS at pH 7.4 with 0.15M NaCl	19.3mV vs. commercial Ag/AgCl in 3M KCl	<12mV in 30h.	2006	[69]
Ag/AgCl	N/A	Photolithography, Lift-off, Sputtering, Electrochemical oxidation	Saturated KCl in Agarose supporting gel.	10mM KCl, pH range 4–10	0.45mV vs. commercial Ag/AgCl	<1.5mV in 42h.	2002	[34]
Ag/AgCl	2cm×0.1 mm	Electroless plating on glass, Electroplating	None (quasi-RE)	3M KCl	13.5mV vs. commercial Ag/AgCl	<30mV in 14 days	2006	[67]
Ag/AgCl	10^6^ μm^2^	Photolithography, Chemical oxidation	None (quasi-RE)	1mM KCl	32mV vs. commercial Ag/AgCl at 1mM KCl, with identical electrode variation of 10mV	<2mV in 5000s	2006	[48]
Ag/AgCl	1μm diameter capillary	Pre-made capillary and silver wire. Capillary action for salt bridge and filling solution	3.3M KCl + AgCl	Distilled water	(−4–0) mV vs. commercial Ag/AgCl	<2 mV in 2400s	2005	[1]
Ag/AgCl	1.2mm^2^	Photolithography, Lift-off, Plasma chlorination	None (quasi-RE)	PBS (0.1M Na_2_HPO_4_, 0.15M NaCl, 0.1 g/l NaN_3_ at pH 7.4)	70mV vs. commercial Ag/AgCl electrode in 3M KCl	<13mV in 5h	2003	[90]
Ag/AgCl	10mm×20mm	Screen-printing thick film	None (quasi-RE)	Technical buffer with 0.05mM Cl^−^	≈230mV vs. commercial Ag/AgCl	<70mV in 12h	2001	[91]
Ag/AgCl	2mm×1.8mm (exposed area only)	Sputtering, Ni layer added, Photolithography, Chemical chloridizing	None (quasi-RE)	50mM Tris buffer (pH 7.4) with 3.5M KCl	0mV vs. commercial Ag/AgCl	<1mV in 2h	2004	[92]
Graphite/AgCl	N/A (macroscopic)	None (macroscopic PVC encasing used)	None (quasi-RE)	KCl 0.1M	40.8mV vs. commercial AgCl in saturated KCl	<0.2mV in 1h	2005	[53]
Hydrogen	4mm×7mm	Evaporation, Lift-off, Galvanic platenization of Pt	None. One variant uses pHEMA membrane	KCl 1M	−850mV vs. fabricated pseudo-Ag/AgCl (after 120–1100 s of initialization drifts)	<1.5mV/h	2000	[85]
Ag/AgI	1mm diameter	Sputtering, Lift-off, PECVD, RIE, Electrodeposition	None (quasi-RE)	PBS, pH 7.0	−94mV vs. SCE	<1mV in 20h	2005	[73]
IrOx	0.1mm×1mm	Photolithography, Sputtering, CVD, Electrodeposition	None (quasi-RE)	PBS	195mV vs. Ag/AgCl	<20mV in 9 days, after initial 120 mV/day drift. 4mV std. dev.	2003	[30]
IrOx	1500μm^2^	Sputtering, Anodic growth (Electrochemical deposition)	None (quasi-RE)	0.1M PBS	40mV vs. Ag/AgCl	<100mV in 15 days	2005	[29]

## References

[1] Suzuki H. (2000). Advances in microfabrication of electrochemical sensors and systems. Electroanalysis.

[2] Ravindra N., Prodan M.C., Fnu S., Padron I., Sikha S.K. (2007). Advances in the manufacturing, types, and applications of biosensors. JOM.

[3] Suzuki H. (2000). Microfabrication of chemical sensors and biosensors for environmental monitoring. Mater. Sci. Eng. C.

[4] Koudelka-Hep M., van der Wal P. (2000). Microelectrode sensors for biomedical and environmental applications. Electrochim. Acta.

[5] Brezinski D.P. (1983). Kinetic, static and stirring errors of liquid junction reference electrodes. The Analyst.

[6] Sharma M. Electrodes and Electrolytes. http://www.physics.usyd.edu.au/super/life_sciences/electricity.html/.

[7] Sevilla F., Kullick T., Scheper T. (1994). A bio-FET sensor for lactose based on co-immobilized β-galactosidase/glucose dehydrogenase. Biosens. Bioelectron.

[8] Shinwari M.W., Deen M.J., Landheer D. (2007). Study of the electrolyte-insulator-semiconductor field-effect transistor (EISFET) with applications in biosensoe design. Microelectron. Reliab.

[9] Benjamin H., Bhansali S., Hoath S.B., Pickens W.L., Smallwood R. (2005). A planar micro-sensor for bio-impedance measurements. Sens. Actuat. B.

[10] Duan Y.Y., Millard R.E., Tykocinski M., Lui X., Clark G.M., Cowan R. (2001). Potential applications of a small and high surface area platinum electrode as an implanted impedance bio-sensor or recording electrode. Proc. SPIE.

[11] Milazzo G., Caroli S., Sharma V.K. (1978). Tables of Standard Electrode Potentials.

[12] Bott A.W. (1996). Mass Transport. Curr. Sep.

[13] Bockris J., Reddy A., Gamboa-Aldeco M. (2000). Modern Electrochemistry 2A: Fundamentals of Electrodics.

[14] Roberge P.R. (2000). Handbook of Corrosion Engineering.

[15] Jayalakshmi V., Ramaswamy R. (1997). Influence of working electrodes in Belousov-Zhabotinsky oscillatory system. Can. J. Chem.

[16] Dorta-Rodríguez R., Barrera-Niebla M., González S., Hernández-Luis F. (1997). Calculation of liquid junction potentials. J. Electroanal. Chem.

[17] Bass L. (1964). Potential of liquid junctions. Trans. Faraday Soc.

[18] Elzanowska H., Birss V. (1993). I. Reversible ageing of iridium oxide electrodes in acidic solutions. J. Appl. Electrochem.

[19] Yu P., Dong S. (1996). The fabrication and performance of an Ag/AgCl reference electrode in human serum. Anal. Chim. Acta.

[20] Shibata M., Siegfried B., Huston J.P. (1977). Miniature calomel electrode for recording DC potential changes accompanying spreading depression in the freely moving rat. Physiol. Behav.

[21] Bockris J.O’M., Devanathan M.A.V., Reddy A.K.N. (1964). The anodic formation of calomel films on mercury electrodes-an ellipsometric-galvanostatic study. Proc. R. Soc. Lond. A.

[22] Lill K.A., Hassel A.W. (2006). A combined μ-mercury reference electrode/Au counter-electrode system for microelectrochemical applications. J. Solid State Electrochem.

[23] Dohner R.E., Wegmann D., Morf W.E., Simon W. (1986). Reference electrode with free-flowing free-diffusion liquid junction. Anal. Chem.

[24] Reiss H., Heller A. (1985). The absolute potential of the standard hydrogen electrode: a new estimate. J. Phys. Chem.

[25] Kita H. (1966). Periodic variation of exchange current density of hydrogen electrode reaction with atomic number and reaction mechanism. J. Electrochem. Soc.

[26] Kita H. (1966). Periodic variation of exchange current density of hydrogen electrode reaction with atomic number and reaction mechanism. Trans. Symp. Elec. Proc..

[27] Appelby A.J., Chemla M., Kita H., Bronoel G. (1982). Encyclopedia of the Electrochemistry of Elements.

[28] Backholm J. (2008). Electrochromic properties of iridium oxide based thin films.

[29] Franklin R.K., Johnson M.D., Scottt K.A., Shim J.H., Nam H., Kipket D.R., Brown R.B. (2005). Iridium oxide reference electrodes for neurochemical sensing with MEMS microelectrode arrays. IEEE Sensors.

[30] Yang H., Kang S.K., Choi C.A., Kim H., Shin D.H., Kim Y.S., Kim Y.T. (2004). An iridium oxide reference electrode for use in microfabricated biosensors and biochips. Lab Chip.

[31] Wang M., Yao S., Madou M. (2002). A long-term stable iridium oxide pH electrode. Sens. Actuat. B.

[32] Goffe R.A., Tseung A.C. (1978). Internally charged palladium hydride reference electrode-Part 1 : The effect of charging current density on long-term stability. Med. & Biol. Eng. & Comput.

[33] Imokawa T., Williams K-J., Denuault G. (2006). Fabrication and Characterization of Nanostructured Pd Hydride pH Microelectrodes. Anal. Chem.

[34] Polk B.J., Stelzenmuller A., Mijares G., MacCrehan W., Gaitan M. (2006). Ag/AgCl microelectrodes with improved stability for microfluidics. Sens. Actuat. B.

[35] Simonis A., Dawgul M., Lüth H., Schöning M.J. (2005). Miniaturised reference electrodes for field-effect sensors compatible to silicon chip technology. Electrochim. Acta.

[36] Broadley S.T., Ragsdale S.R., Silverman H.P. (2003). Reference electrode having a microfluidic flowing liquid junction.

[37] Boodts J.C.F., Trasatti S. (1989). Hydrogen evolution on iridium oxide cathodes. J. Appl. Electrochem.

[38] McGill I.R., McEnanay B. (1978). A novel reference electrode arrangement for high temperature polarisation studies. Corros. Sci.

[39] Garbett K., Torrance K. (1975). Capillary tip reference electrode based on a glass syringe [for metal surface corrosion measurement]. Lab. Pract..

[40] Savinell R.F., Liu C.C., Kowalsky T.E., Puschett J.B. (1981). Miniature Glass pH Electrode with Nonaqueous Internal Reference Solution. Anal. Chem.

[41] Kitade T., KITAMURA K., Takegami S., Miyata Y., Nagatomo M., Sakaguchi T., Furukawa M. (2005). Needle-type ultra micro silver/silver chloride reference electrode for use in micro-electrochemistry. Anal. Sci.

[42] Zhang X., Ogorevc B., Tavčar G., Švegl I.G. (1996). Over-oxidized polypyrrole-modified carbon fibre ultramicroelectrode with an integrated silver/silver chloride reference electrode for the selective voltammetric measurement of dopamine in extremely small sample volumes. Analyst.

[43] Pedrotti J.J., Angnes L., Gutz I.G.R. (1996). Miniaturized reference electrodes with microporous polymer junctions. Electroanalysis.

[44] Czaban J.D., Rechnitz G.A. (1976). Glass Microelectrode Probes for Routine pH Measurements. Anal. Chem.

[45] Jin X., Lu J., Liu P., Tong H. (2003). The electrochemical formation and reduction of a thick AgCl deposition layer on a silver substrate. J. Electroanal. Chem.

[46] Jović B.M., Jović V.D., Dražić D.M. (1995). Kinetics of chloride ion adsorption and the mechanism of AgCl layer formation on the (111), (100) and (110) faces of silver. J. Electroanal. Chem.

[47] Eine K., Kjelstrup S., Nagy K., Syverud K. (1997). Towards a solid state reference electrode. Sens. Actuat. B.

[48] Huang I-Y, Huang R-S, Lo L-H. (2002). Fabrication and characterization of a new planar solid-state reference electrode for ISFET sensors. Thin Solid Films.

[49] Liao W-Y, Chou T-C (2006). Fabrication of a planar-form screen printed solid electrolyte modified Ag/AgCl reference electrode for application in a potentiometric biosensor. Anal. Chem.

[50] Mamińska R., Dybko A., Wróblewski W. (2006). All-solid-state miniaturised planar reference electrodes based on ionic liquids. Sens. Actuat. B.

[51] Zielińska R., Mulik E., Michalska A., Achmatowicz S., Maj-Żurawska M. (2002). All-solid-state planar miniature ion-selective chloride electrode. Anal. Chim. Acta.

[52] Yun K-S, Kim H-J, Joo S., Kwak J., Yoon E (2000). Analysis of heavy-metal ions using mercury microelectrodes and a solid-state reference electrode on a Si wafer. Jpn. J. Appl. Phys.

[53] Valdés-Ramírez G., Álvarez-Romero G., Galán-Vidal C.A., Hernández-Rodríguez P.R., Ramírez-Silva M. (2005). Composites: a novel alternative to construct solid-state Ag/AgCl reference electrodes. Sens. Actuat. B.

[54] Nagy K., Eine K., Syverud K., Aune O. (1997). Promising new solid-state reference electrode. J. Electrochem. Soc.

[55] Nolan M.A., Tan S.H., Kounaves S.P. (1997). Fabrication and characterization of a solid state reference electrode for electroanalysis of natural waters with ultramicroelectrodes. Anal. Chem.

[56] Moussy F., Harrison D.J. (1999). Prevention of the rapid degradation of subcutaneously implanted Ag/AgCl reference electrodes using polymer coatings. Anal. Chem.

[57] Kwon N-H, Lee K-S, Won M-S, Shim Y-B (2007). An all-solid-state reference electrode based on the layer-by-layer polyumer coating. Analyst.

[58] Tymecki L., Zwierkowska E., Koncki R. (2004). Screen-printed reference electrodes for potentiometric measurements. Anal. Chim. Acta.

[59] Smith R.L, Scott D.C. (1984). A solid state miniature reference electrode.

[60] Smith R.L, Scott D.C. (1986). Miniature liquid junction reference electrode and an integrated solid state electrochemical sensor including the same.

[61] Yee S., Jin H., Lam L.K.C. (1988). Miniature liquid junction reference electrode with micromachined silicon cavity. Sens. Actuat.

[62] van den Berg A., Grisel A., van den Vlekkert H.H., de Rooij N.F. (1990). A micro-volume open liquid-junction reference electrode for pH-ISFETs. Sens. Actuat. B.

[63] Sinsabaugh S.L., Fu C.W., Fung C.D. (1986). A batch-processed reference micro electrode integrated on a silicon substrate. Proc. Electrochem. Soc.

[64] Suzuki H., Hiratsuka A., Sasaki S., Karube I. (1998). Problems associated with the thin-film Ag/AgCl reference electrode and a novel structure with improved durability. Sens. Actuat. B.

[65] Suzuki H., Hirakawa T., Sasaki S., Karube I. (1998). Micromachined liquid-junction Ag/AgCl reference electrode. Sens. Actuat. B.

[66] Suzuki H., Shiroishi H., Sasaki S., Karube I. (1999). Microfabricated liquid junction Ag/AgCl reference electrode and its application to a one-chip potentiometric sensor. Anal. Chem.

[67] Sun X., Wang M. (2006). Fabrication and characterization of planar reference electrode for on-chip electroanalysis. Electrochim. Acta.

[68] Yalcinkaya F., Powner E.T. (1997). Ag/AgCl/Cl^−^ coated silver stripe reference electrode. Med. Eng. Phys.

[69] Kim S.K., Lim H., Chung T.D., Kim H.C. (2006). A miniaturized electrochemical system with a novel polyelectrolyte reference electrode and its application to thin layer electroanalysis. Sens. Actuat. B.

[70] Tymecki Ł., Zwierkowska E., Koncki R. (2004). Screen-printed reference electrodes for potentiometric measurements. Anal. Chim. Acta.

[71] Simonis A., Lüth H., Wang J., Schöning M.J. (2004). New concepts of miniaturised reference electrodes in silicon technology for potentiometric sensor systems. Sens. Actuat. B.

[72] Ito S., Kobayashi F., Baba K., Asano Y., Wada H. (1996). Development of long-term stable reference electrode with fluoric resin liquid junction. Talanta.

[73] Cunnane V.J., Schiffrin D.J., Williams D.E. (1995). Micro-cavity electrode: a new-type of liquid-liquid microelectrode. Electrochim. Acta.

[74] Rivas I., Puente D., Ayerdi I., Castaño E. Ag/AgI quasi-reference microelectrodes.

[75] Ciobanu M., Wilburn J.P., Lowy D.A. (2004). Miniaturized reference electrodes. II. use in corrosive, biological, and organic media. Electroanalysis.

[76] Thomas R.C., Cohen C.J. (1981). A liquid ion-exchanger alternative to KCl for filling intracellular reference microelectrodes. Pflügers Archiv Eur. J. Physiol.

[77] Saheb A., Janata J., Josowicz M. (2006). Reference electrode for ionic liquids. Electroanalysis.

[78] Matsumoto T., Ohashi A., Ito N. (2002). Development of a micro-planar Ag/AgCl quasi-reference electrode with long-term stability for an amperometric glucose sensor. Anal. Chim. Acta.

[79] Ferreira H.E.A., Daniel D., Bertotti M., Richter E.M. (2008). A novel disposable electrochemical microcell: construction and characterization. J. Braz. Chem. Soc.

[80] Silva R.A.B., Almeida E.G.N., Rabelo A.C., Silva A.T.C., Ferreira L.F., Richter E.M. (2009). Three electrode electrochemical microfluidic cell: construction and characterization. J. Braz. Chem. Soc.

[81] Yosypchuk B., Novotny L. (2004). Reference electrodes based on solid amalgams. Electroanalysis.

[82] Kunimatsu M., Qiao H., Okada T. (2005). Microtubular hydrogen electrode, a reference electrode for electrochemical analyses. J. Electrochem. Soc.

[83] Will F.G. (1985). A self-contained, miniature hydrogen reference electrode. J. Electrochem. Soc.

[84] Gong S., Lu J., Yan H. (1997). Developing the self-contained hydrogen reference electrode. J. Electoanal. Chem.

[85] Nann T., Urban G.A. (2000). A new dynamic hydrogen reference electrode for applications in thin-film sensor systems. Sens. Actuat. B.

[86] Giner J. (1964). A practical reference electrode. J. Electrochem. Soc.

[87] Keller O.C., Buffle J. (2000). Voltammetric and reference microelectrodes with integrated microchannels for flow through microvoltammetry. 1. The microcell. Anal. Chem.

[88] Wipf D.O., Ge F., Spaine T.W., Baur J.E. (2000). Microscopic measurement of pH with iridium oxide microelectrode. Anal. Chem.

[89] Yang H., Kang S.K., Shin D-H., Kim H., Kim Y.T. Microfabricated iridium oxide reference electrode for continuous glucose monitoring sensor.

[90] Park S-I., Jun S.B., Park S., Kim H.C., Kim S.J. (2003). Application of a new Cl-plasma-treated Ag/AgCl reference electrode to micromachined glucose sensor. IEEE Sens. J.

[91] Simonis A., Krings T., Lüth H., Wang J., Schöning M.J. (2001). A “hybrid” thin-film pH sensor with integrated thick-film reference. Sensors 2001.

[92] Kim H.R., Kim Y.D., Kim K.I., Shim J.H., Nam H., Kang B.K. (2004). Enhancement of physical and chemical properties of thin film Ag/AgCl reference electrode using a Ni buffer layer. Sens. Actuat. B.

[93] Bergstorm P.L., Patel S.V., Schwank J.W., Wise K.D. (1997). A micromachined surface work-function gas sensor for low-pressure oxygen detection. Sens. Actuat. B.

[94] Gergintschew Z, Kornetzky P., Schipanski D. (1996). The capacitively controlled field effect transistor (CCFET). Sens. Actuat. B.

[95] Fedirko N., Svichar N., Chesler M. (2006). Fabrication and use of high-speed, concentric H^+^ - and Ca^2+^ -selective microelectrodes suitable for in vitro extracellular recording. J. Neurophysiology.

[96] Kurkdijan A.C., Barbier-Brygoo H. (1983). A hydrogen ion-selective liquid-membrane microelectrode for measurement of the vacuolar pH of plant cells in suspension culture. Anal. Biochem.

[97] Zine N., Bausells J., Ivorra A., Aguilo J., Zabala M., Teixidor F., Masalles C., Vinas C., Errachid A. (2003). Hydrogen-selective microelectrodes based on silicon needles. Sens. Actuat. B.

[98] Chao P., Ammann D., Oesch U., Simon W., Lang F. (1988). Extra- and intracellular hydrogen ion-selective microelectrode based on neutral carriers with extended pH response range in acid media. Pflügers Arch. European J. Physiol.

[99] Koryta J. (1986). Ion-selective electrodes. Ann. Rev. Mat. Sci.

[100] Covington A.K., Bates R.G., Durst R.A. (1985). Definition of pH scales, standard reference values, measurement of pH and related terminology. Pure Appl. Chem.

[101] Lengyel B., Blum E. (1934). The behaviour of the glass electrode in connection with its chemical composition. Trans. Faraday Soc.

[102] Kerridge P.T. (1925). The use of the glass electrode in biochemistry. Biochem. J.

[103] Vanýsek P. (2004). The glass pH electrode. Interface-The Electrochem. Soc. Pub.

[104] Gooding J., Hibbert D., Yang W. (2001). Electrochemical metal ion sensors. Exploiting amino acids and peptides as recognition elements. Sensors.

[105] Eisenman G. (1962). Cation selective glass electrodes and their mode of operation. Emerging Techniques in Biophysics.

[106] Ikeda T., Hamada H., Miki K., Senda M. (1985). Glucose oxidase-immobilized benzoquinone- carbon paste electrode as glucose sensor. Agric. Biol. Chem.

[107] Wang J., Walcarius A. (1996). Zeolite-modified carbon paste electrode for selective monitoring of dopamine. J. Electroanal. Chem.

[108] Guan W-J, Li Y., Chen Y-Q, Zhang X-B, Hu G-Q (2005). Glucose biosensor based on multi-wall carbon nanotubes and screen printed carbon electrodes. Biosens. Bioelectron.

[109] Zhang X., Wang J., Ogorevc B., Spichiger U.E. (1999). Glucose nanosensor based on prussian-blue modified carbon-fiber cone nanoelectrode and an integrated reference electrode. Electroanalysis.

[110] Hiratsuka A., Kojima K-I., Suzuki H., Muguruma H., Ikebukuro K., Karube I. (2001). Integration of microfabricated needle-type glucose sensor devices with a novel thin-film Ag/AgCl electrode and plasma-polymerized thin film: mass production techniques. The Analyst.

[111] Patolsky F., Zayats M., Katz E., Willner I. (1999). Precipitation of an insoluble product on enzyme monolayer electrodes for biosensor applications: characterization by faradaic impedance spectroscopy, cyclic voltammetry, and microgravimetric quartz crystal microbalance analyses. Anal. Chem.

[112] Cui G., Lee J.S., Kim S.J., Nam H., Cha G.S., Kim H.D. (1998). Potentiometric pCO_2_ sensor using polyaniline-coated pH-sensitive electrodes. Analyst.

[113] Suzuki H., Kojima N., Sugama A., Fujita S. (1991). Micromachined clark oxygen electrode.

[114] Suzuki H., Hirakawa T., Sasaki S., Karube I. (2000). An integrated module for sensing pO_2_, pCO_2_, and pH. Anal. Chim. Acta.

[115] Souteyrand E, Cloarec J.P., Martin J.R., Wilson C., Lawrence I., Mikkelsen S., Lawrence M.F. (1997). Direct detection of the hybridization of synthetic homo-oligomer DNA sequences by the field-effect. J. Phys. Chem. B.

[116] Ingebrandt S., Offenhäusser A. (2006). Label-free detection of DNA using field-effect transistors. Phys. Status Solidi A.

[117] Kim D.-S., Park H.-J., Jung H.M., Shin J.K., Choi P., Lee J.H., Lim G. (2004). Field effect transistor-based bimolecular sensor employing a Pt reference electrode for the detection of deoxyribonucleic acid sequence. Jpn. J. Appl. Phys.

[118] Park K-M, Lee S-K, Sohn Y-S, Choi S-Y (2008). BioFET sensor for detection of albumin in urine. Electron. Lett.

[119] Minot E.D., Janssens A.M., Heller I., Heering H.A., Dekker C., Lemay S.G. (2007). Carbon nanotube biosensors: the critical role of the reference electrode. Appl. Phys. Lett.

[120] Jamasb S., Collins S., Smith R.L. (1998). A physical model for drift in pH ISFETs. Sens. Actuat. B.

[121] Jamasb S. (2004). An analytical technique for counteracting drift in ion-selective field effect transistors. IEEE Sens. J.

[122] Jamasb S, Collins S.D., Smith R.L. (1997). Correction of instability in ion-selective field-effect transistors (ISFET’s) for accurate continuous monitoring of pH.

[123] Mascini M., Palchetti I., Marrazza G. (2001). DNA electrochemical biosensors. Fresenius J. Anal. Chem.

[124] Brett C.M.A., Inzelt G., Kertesz V. (1999). Poly(methylene blue) modified electrode sensor for haemoglobin. Anal. Chim. Acta.

[125] Schwarz M.A., Galliker B., Fluri K., Kappes T., Hauser P.C. (2001). A two-electrode configuration for simplified amperometric detection in a microfabricated electrophoretic separation device. Analyst.

[126] Safari-Mohsenabad Salman, Selvaganapathy P.R., Deen M.J. Surface micromachined PDMS microfluidic devices fabricated using a sacrificial photoresist.

[127] Safari-Mohsenabad Salman, Selvaganapathy P.R., Deen M.J. (2010). Microfluidic Reference Electrode with Flowing Liquid Junction.

[128] Safari-Mohsenabad Salman, Selvaganapathy P.R., Derardja A., Deen M.J. (2010). Nanosheet formation by modified electrodeposition method and its application to miniaturized reference electrodes.

[129] Shinwari M.W, Deen M.J., Selvaganapathy P.R. (2008). Analytic Modeling of Biotransistors. IET Circuits, Devices & Systems.

[130] Deen M.J., Shinwari M.W., Ranuárez J.C., Landheer D. (2006). Noise Considerations in Field-Effect Biosensors. J. Appl. Phys.

[131] Landheer D., Aers G., McKinnon W.R., Deen M.J., Ranuarez J.C. (2005). Model for the Field-Effect from Layers of Biological Macromolecules on the Gates of Metal-Oxide-Semiconductor Transistors. J. Appl. Phys.

[132] Bustillo J.M., Howe R.T., Muller R.S. (1998). Surface micromachining for microelectromechanical systems. Proc. IEEE.

[133] Subramani B.G., Selvaganapathy P.R. (2009). Surface micromachined PDMS microfluidic devices fabricated using a sacrificial photoresist. J. Micromech. Microeng.

